# Supplementary material for: The Evolution and Characterization of the RNA Interference Pathways in Lophotrochozoa
Source: Genome Biol Evol. 2024 May 7;16(5):evae098. doi: 10.1093/gbe/evae098 (PMC11114477; doi:10.1093/gbe/evae098)
Supplement: evae098_Supplementary_Data [file evae098_supplementary_data.zip › Supplementary_materials.zip!/selected_miRNAs/DaRe_selected_miRNas/DaRe_NC_007119.7_20507.pdf]

Provisional ID	:	NC_007119.7_20507
Score total	:	33.1
Score for star read(s)	:	3.9
Score for read counts	:	26.6
Score for mfe	:	1.6
Score for randfold	:	1.6
Score for cons. seed	:	-0.6
Total read count	:	64
Mature read count	:	46
Loop read count	:	0
Star read count	:	18

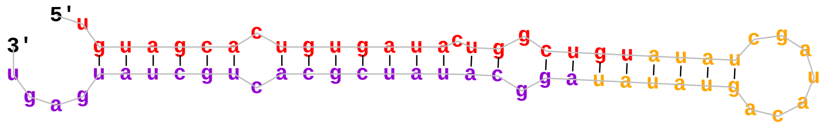

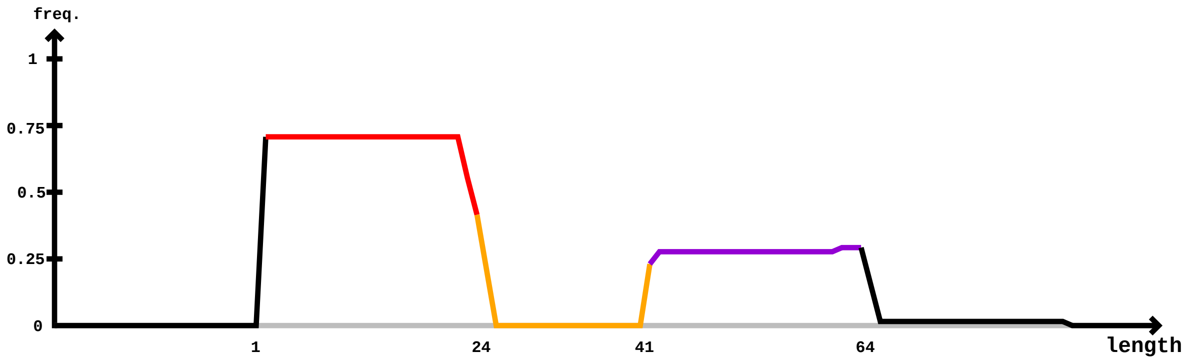

Mature Star

```
5'- uaaaugagcaauaucacauauguagcacugugauacuggcuguauaucgauacaguauauaggcauaucgcacugcuaugaguguuauauugcguuuauacaacaguucgaca  -3'   obs
    uaaaugagcaauaucacauauguagcacugugauacuggcuguauaucgauacaguauauaggcauaucgcacugcuaugaguguuauauugcguuuauacaacaguucgaca         exp
    .......(((((((.((((.(((((((.(((((((.((.((((((((.......)))))))).))))))))).))))))).)))).)))))))...................   reads   mm   sample
    ............................................................aggcauaucgcacugcuaugagu..............................   1       0    Ov2

    ....................uguagcacugugauacuggcu........................................................................   4       0    Gu3
    ....................uguagcacugugauacuggcuA.......................................................................   1       1    Gu3
    ....................uguagcacugugauacuggcugu......................................................................   5       0    Gu3
    ............................................................aggcauaucgcacugcuaugagu..............................   2       0    Gu3
    ............................................................aggcauaucgcacugcuaugagA..............................   1       1    Gu3
    .............................................................ggcauaucgcacugcuaugagu..............................   2       0    Gu3
    ................................................................................aguguGauauugcguuuauacaac.........   1       1    Gu3

    ....................uguagcacugugauacuggcug.......................................................................   1       0    Ov1
    ....................uguagcacugugauacuggcugu......................................................................   2       0    Ov1

    ....................uguagcacugugauacuggcu........................................................................   1       0    Gu1
    ....................uguagcacugugauacuggcug.......................................................................   2       0    Gu1
    ....................uguagcacugugauacuggcugu......................................................................   8       0    Gu1
    ............................................................aggcauaucgcacugcuaugagu..............................   6       0    Gu1
    .............................................................ggcauaucgcacugcuaugagu..............................   1       0    Gu1

    ....................uguagcacugugauacuggcu........................................................................   1       0    Ov3
    ....................uguagcacugugauacuggcugu......................................................................   1       0    Ov3
    ............................................................aggcauaucgcacugcuaugagu..............................   1       0    Ov3

    ....................uguagcacugugauacuggcu........................................................................   4       0    Gu2
    ....................uguagcacugugauacuggcug.......................................................................   4       0    Gu2
    ....................uguagcacugugauacuggcuA.......................................................................   1       1    Gu2
    ....................uAuagcacugugauacuggcugu......................................................................   1       1    Gu2
    ....................uguagcacugugauacuggcugu......................................................................   9       0    Gu2
    ....................uguagcacugugauacuggcuguU.....................................................................   1       1    Gu2
    ............................................................aggcauaucgcacugcuaugagu..............................   4       0    Gu2
```
